# Developing an Effective Community Oral Health Workers—“Promotoras” Model for Early Head Start

**DOI:** 10.3389/fpubh.2019.00175

**Published:** 2019-07-03

**Authors:** Jennifer Villalta, Hamida Askaryar, Inese Verzemnieks, Janni Kinsler, Vickie Kropenske, Francisco Ramos-Gomez

**Affiliations:** ^1^School of Dentistry, University of California, Los Angeles, Los Angeles, CA, United States; ^2^School of Nursing, University of California, Los Angeles, Los Angeles, CA, United States; ^3^Section of Pediatric Dentistry, University of California, Los Angeles, Los Angeles, CA, United States; ^4^School of Public Health, University of California, Los Angeles, Los Angeles, CA, United States

**Keywords:** Early Childhood Caries (ECC), prevention, Community Health Workers (CHWs), promotoras de salud, workforce development

## Abstract

**Purpose:** To determine the effectiveness of a train-the-trainer program for Community Oral Health Workers (COHWs) with the goal of reducing Early Childhood Caries (ECC).

**Methods:** Thirteen Latina caregivers from a local Early Head Start program participated in an 8 h bilingual oral health training program that provided information and hands-on experiences pertaining to prenatal and children's oral health. Once trained, the 13 COHWs conducted a series of bilingual interactive oral health promotion workshops at local community sites. Pre/post-tests were conducted after each workshop with a total of 157 caregivers of young children. Bivariate analyses were used to assess changes in knowledge, attitudes, and practices of the COHWs and caregivers regarding children's oral health.

**Results:** Significant positive changes (*p* < 0.05) in COHWs' knowledge were observed for age a child can brush his/her teeth alone and what a pregnant woman with morning sickness can do to protect her teeth. Positive trends were observed for knowing that tap water with fluoride prevents cavities and that poor oral health of parents affects their children's dental health. While community caregivers in the workshops reported a high consumption of sweet snacks and beverages, there was a significant positive increase (*p* < 0.05) in knowledge and attitudes regarding oral health care. Significant increases in knowledge were obtained regarding: when a child can brush his/her teeth well alone, the age when fluoridated toothpaste can be used, ways tooth decay can be prevented, when a child's first dental visit should be, and what a pregnant woman with morning sickness can do to protect her teeth. Significant positive improvements were found regarding caregiver's favorable attitude that fluoridated water can help prevent cavities, disagreeing that tap water is dangerous, and agreeing that a parent's dental health affects their children's dental health.

**Conclusions:** The study showed a targeted and culturally competent oral health program can significantly improve knowledge, attitudes, and self-reported practices of COHWs and the caregivers they trained. Although longitudinal studies are needed to determine if a COHW model can help reduce ECC in underserved communities, preliminary results support the utilization of this model as a viable option that should be expanded.

## Introduction

Dental caries is the most common chronic childhood infectious disease and continues to be a serious public health problem affecting both developing and industrialized countries, yet it is preventable (1–6). In the U. S., Early Childhood Caries (ECC) affects 53.8% of children ages 2–19 years and ~12% of children ages 2–5 years (7, 8). Children from disadvantaged populations and low socioeconomic status are at increased risk for developing ECC. For example, among youth 2–19 years of age, total dental caries affects Hispanics (57.1%) disproportionally as compared to their non-Hispanic Black (48.1%) and non-Hispanic White (40.4%) counterparts (7).

Additionally, the proportion of children with *untreated* dental caries in their primary teeth increases with age: 10.9% among children aged 2–5 years and 17.4% among children aged 6–8 years; a larger proportion of Hispanic (19.4%) and non-Hispanic black children (19.3%) had *untreated* dental caries in primary teeth compared with non-Hispanic white (9.5%) children (8). Left untreated, dental caries can lead to infections, pain and loss of teeth, affecting the child's quality of life by interfering with the ability to perform daily activities like eating, sleeping, learning, and playing (9–11). From 2011 to 2016, a decrease was seen in the prevalence of total dental caries (from 50.0 to 45.8%) in youth, however, this decline was not statistically significant (7).

While numerous factors contribute to ECC (diet, oral hygiene habits, oral bacteria, access to care, etc.), it can be preventable with appropriate behavior modifications (6, 12, 13). Although oral health education is not the only step in dental caries prevention, it is a critical and important factor, especially among vulnerable populations who experience less access to oral health care services (9, 14).

The Hispanic/Latino population is projected to be the third fastest growing minority group in the U.S., which is estimated to increase by 115% by 2060 (15). Thus, dentistry's success in meeting the challenges of an increasingly ethnically diverse population is dependent upon considering the larger social and cultural context in order to have an impact on personal behavior change (9, 16–18). Community Health Workers (CHW) play a vital role in connecting marginalized and medically underserved populations to the health and social service systems intended to serve them (19–22). CHWs (i.e., promotoras, lay-person health educators, peer health advocates) work in community settings and serve as connectors between health care consumers and providers to promote health among groups that lack access to adequate care (16, 23, 24). CHW's understand the cultural perspectives of the communities they serve and can help reduce barriers to health care and increase access to preventive oral health services (16, 19).

The “promotora” CHW model is a culturally appropriate approach to delivering preventive health interventions for Latino populations, and has been shown to be effective in creating behavior change in reducing chronic disease risk factors and health conditions through education, dietary interventions, and increasing screening rates (9, 19, 25–29). Promotoras have been used extensively to reach diverse populations such as mothers and infants, migrant farm workers, and Latino populations. Adams et al. showed significant improvements in plaque index, bleeding on probing, and pocket depths 4 mm or greater after promotora-provided brief oral health education and skill-building activities during prenatal care (30). In the Contra Caries Oral Health Education Program (CCOHEP), promotoras led 2 h interactive sessions for Spanish-speaking parents. Immediately after attending CCOHEP, caregiver-reported behavior performance improved to 44% (from baseline of 13%); 3 months after attendance, it increased to 66%. Four of the five reported tooth brushing behaviors improved between pretest and post-test, especially brushing at night (12). Thus, the CHW promotora model is a promising method for improving oral health-related knowledge and behavior among underserved and underinsured minority communities (9, 31–33).

The purpose of this study was to train Latina Community Oral Health Workers (COHWs) using a promotora CHW model to educate underserved and minority populations in Los Angeles County on best practices in oral health care, with the ultimate goal of reducing ECC. The trained COHWs then conducted oral health workshops in the community for other caregivers of young children. We hypothesize there will be significant increases in knowledge and positive changes in oral health beliefs and practices of the COHWs and caregivers after the oral health training/workshops using pre-/post-tests.

## Methods

The UCLA School of Dentistry through the Center for Children's Oral Health (34) conducted a pilot project in 2016-2017 (COHWs I) with two community partner sites in Los Angeles County. In the COHW I pilot study, 10 Latina caregivers were trained as COHWs. The pre/post-tests of the 10 COHWs showed a significant increase in total knowledge and practices (35). Building on these findings, a Professor of pediatric dentistry and a bilingual Latina pediatric dental resident developed this COHW II project in which caregivers were trained as COHWs. After completion of the oral health training, the COHWs conducted oral health workshops in the community for other caregivers of young children. Changes in the COHWs and caregiver's knowledge, beliefs, and practices regarding children's oral health were assessed before and after the COHW training and the COHW-led workshops.

### Participants and Community Partners

Hope Street Margolis Family Center (HSMFC) is a well-established community center located in downtown Los Angeles (36) which is a health, education, and recreational resource for children and families and also provides the federally funded Early Head Start (EHS) program. EHS supports low-income pregnant women and families with infants and toddlers with in-home and on-site educational, health/wellness, developmental, and social services. Upon consultation with the HSMFC management team, it was decided to make the COHW training open to members of the EHS Policy Council. The Policy Council is a group of EHS parents who are selected by their peers to help advise the Head Start director and governing board on important matters related to the center. These caregivers have already shown leadership potential that could be further developed by their participation in this project. Thirteen EHS parents volunteered to participate in the COHW II training and received $550 for their time and participation, which was given in the form of small gift cards and cash to offset their cost of transportation and childcare.

### Procedures

The project lasted 14 months and was completed in the following three phases:

**Phase I**: Formative research and oral health curriculum development,
**Phase II**: COHW training, and
**Phase III**: COHW-led oral health promotion workshops.

**Figure 1:**
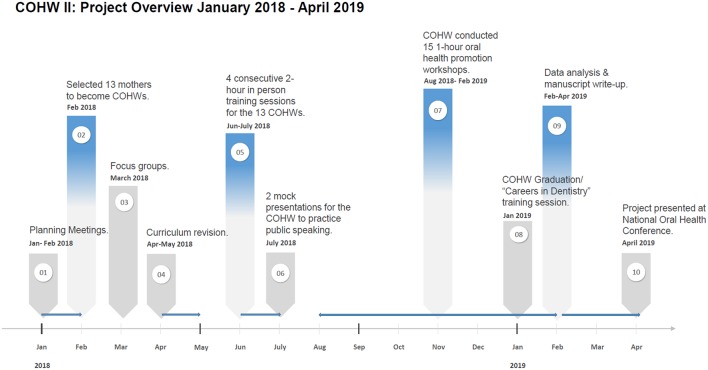
Timeline.

Figure 1 presents the project timeline and overview. All participants gave written informed consent prior to participation and UCLA IRB approval was obtained (**IRB # 18-000014**).

### Phase I: Formative Research (Focus Group) and Oral Health Curriculum Development

A focus group was conducted with Latina caregivers of young children from HSMFC in the third month of the project development with the aim of revising the existing oral health curriculum from COHW I to meet the needs of the target population for the current COHW II study. All caregivers with children between the ages of 0–5 were eligible to participate in the focus group. Bi-lingual recruitment flyers were posted at the HSMFC. The HSMFC family services coordinator assisted with recruitment and coordination of the focus group. The majority of volunteer participants were bilingual (English/Spanish) Latina mothers, between the ages of 20–42 years, had a high school education and were married. The focus group was conducted at HSMFC and was led by a bilingual pediatric dental resident. The focus group lasted 90 min and was guided by open-ended questions on tooth brushing habits, toothpaste usage, fluoridated water usage, dental visits, dental insurance, and barriers to dental care. Notes were taken during the focus group and specific quotes were documented and highlighted. After the focus group, the notes were fully transcribed. Notes and open-ended questions were examined for themes using content analysis. The focus group was not audio-recorded.

Based on focus group findings and consultations with the UCLA interprofessional team of dentists and nurses, the existing COHW I curriculum was revised. The COHW II curriculum for this study included the following five main topic areas:

1. Review of basic oral anatomy and characteristics of healthy vs. unhealthy teeth (using many pictures, tooth models, and videos),
2. Teaching COHWs how to identify differences between normal and abnormal findings in the mouth (e.g., how to look for cavities),
3. Creating awareness of pregnancy and child preventive oral health practices known to reduce ECC (morning sickness, tooth brushing, flossing, snacking, fluoride, etc.),
4. Creating awareness of particular challenges involved in dealing with special needs children such as autistic children (e.g., how to brush the teeth of an uncooperative child and where to seek dental help for special needs children) and
5. How to perform a regular and thorough basic oral screening of infants and children.

The curriculum was kept at a 6th grade literacy level and all written materials were translated (and back translated) into Spanish. The curriculum is available at the following link: http://www.uccoh.org/research.html (34).

### Phase II: COHW Training

The COHW training consisted of 4 consecutive weekly 2 h sessions which included a combination of classroom lectures, hands-on training, and discussions. Total in-person training time was 8 h; an additional 4–5 h for reading assigned homework materials and listening to webinars was required. The training introduced COHWs to evidence-based health knowledge about the nature, prevalence, and consequences of oral manifestations of chronic oral diseases across the lifespan with an emphasis on children as well as the oral-systemic connection. The COHWs learned how to assess a child's oral health, identify basic healthy vs. abnormal oral conditions and to apply basic concepts of a caries risk assessment (37). They performed basic oral health screenings on their own children and children of their peers. They learned their role as promotoras in preventing oral disease in the community, addressing frequently encountered oral health problems, and promoting oral health in their community. Reflection sessions allowed the COHWs and the project team to exchange ideas and thoughts. The COHWs were required to review a list of course materials prior to the start of some of the trainings. These included selected online sections of the “Smiles for Life” curriculum (38), a Colgate Webinar on: “The Art of Perinatal and Infant Oral Health” by Dr. Ramos-Gomez (available in Spanish), and links to the UCLA Infant Oral Care Clinic documents (39). Training sessions were presented simultaneously in English and Spanish by the pediatric dental resident and two nurse practitioner students. The pediatric dental resident conducted sessions 1, 3, and 4 and the two nursing practitioner students conducted session 2. Childcare and light refreshments were available at all training meetings. Table 1 provides a brief description of the topics covered during the 4 training sessions:

**Table 1:** 4 in-person bilingual training sessions (each 2 h) for the 13 COHWs.

**Training session 1**	**Training session 2**	**Training session 3**	**Training session 4**
• Introduction to basic oral health • Early Childhood Caries (ECC) development • Caries risk & protective factors • Prevention tips for all ages	• Oral health care during pregnancy • Pregnancy gingivitis • Morning sickness treatment • Tap water • Nutrition	• Nutrition and snacking • Brushing and flossing • White Spot Lesions (WSL) • Caries progression • Fluoride • Teething	• Radiographs • Types of child dental treatments • Emergency dental care • Nitrous oxide vs. oral sedation vs. general anesthesia • Insurance • Self-management goals • Motivational Interviewing

### Phase III: COHW-Led Oral Health Promotion Workshops

The 13 COHWs who completed the training conducted 15 1 h bilingual oral health promotion workshops in teams of two to a convenience sample of 157 caregivers of young children in the local community. These 1 h bilingual oral health workshops were conducted at local elementary schools, parks, homes, and WIC sites. A UCLA team member was present to assist and help collect data. The bilingual pediatric dentist attended most workshops, offering support. Workshop locations, dates, and times were selected by the COHWs who made all the necessary arrangements (flyers, number of anticipated attendees, etc.) with the host site. UCLA provided free oral hygiene supplies, small raffle gifts, and light refreshments at all workshops. The curriculum used for these workshops was a condensed version of the curriculum used for the COHW training. All written material was kept at a 6th grade literacy level and was translated (and back translated) into Spanish. The learning objectives for the 1 h oral health workshops conducted by the COHWs are listed below:

1. To understand the importance of perinatal oral health care, including vertical transmission.
2. To be able to list the main causes of Early Childhood Caries (ECC).
3. To be able to list the negative impacts of ECC.
4. To identify the early signs of caries.
5. To understand how to prevent ECC through the use of fluoride, dental home by age 1, proper oral hygiene, and appropriate nutrition.
6. To identify the benefits of fluoridated tap water.
7. To demonstrate the proper brushing and flossing techniques.

### Data Collection

The 13 COHWs who participated in the training completed a 34-item written paper and pencil pre- and post-test questionnaire (available in English and Spanish) to assess changes in knowledge, beliefs, and practices regarding children's oral health (see Supplementary Datasheet 1 for the COHW questionnaire). Fourteen items were related to oral health knowledge, 10 addressed the COHWs beliefs toward children's oral health, and 10 related to oral health practices. Answer choices ranged from multiple choice, true/false, Likert scales (strongly agree/agree/disagree/strongly disagree), and some write-in answers. The pre-test was completed by the COHWs at the beginning of the first training session. The post-test was completed 6 weeks after the training ended.

Caregivers participating in the workshops completed a written paper and pencil pre- and post-test questionnaire (it took between 8 and 10 min to fill it out) to assess changes in knowledge and beliefs regarding children's oral health. Pre-test questionnaires were filled out at the beginning of each workshop and the post-tests were completed immediately following. The questionnaire contained 16 items on the pre-test and 11 items on the post-test (see Supplementary Datasheet 2, 3 for the caregiver questionnaire). Pre- and post-test responses were matched. Eight items related to oral health knowledge, three addressed beliefs about children's oral health, and five were related to oral health practices. Oral health practices were only addressed on the pretest as changes in practice would not be measureable following a 1 h workshop. Answer choices included multiple choice, true/false, Likert scales (strongly agree/agree/disagree/ strongly disagree), and some write-in answers.

### Statistical Analysis

Data for the COHW training and caregiver workshops were entered into RedCap and analyzed with R software (www.r-project.com). Summary statistics were generated to characterize the COHWs (*N* = 13) and the workshop attendees (*N* = 157). Paired *t*-tests and McNemar tests were used to assess changes in knowledge, beliefs, and practices between pre- and post-test results for the COHW and changes in knowledge and beliefs for workshop attendees.

## Results

### Phase I: Formative Research (Focus Group)

Four major topic areas regarding oral health care were identified among focus group participants: (1) tooth brushing habits, (2) knowledge regarding cavities and baby teeth, (3) drinking water/fluoridation, and (4) barriers to dental care. Below is a summary of the findings along with representative quotes for each topic area.

### Tooth Brushing Habits

Common tooth brushing habits among children included brushing in the morning and evening and brushing after their child eats candy. The two biggest factors affecting tooth brushing were bad taste of toothpaste and children being scared of the toothbrush. Singing and playing music were reported as strategies for calming children down so parents could brush their teeth.

“*Every time my child wakes up, she brushes. She also brushes at night time.”*

“*My child brushes first and then I check or brush for her.”*

### Knowledge Regarding Cavities and Baby Teeth

Focus group participants described cavities as being black stains, spots, or holes. The most common responses regarding causes of cavities were drinking milk (especially during night if a child sleeps with a bottle), mother giving cavities to their child, and not brushing your child's teeth consistently. Most participants knew that cavities in baby teeth could affect adult teeth. If cavities were not treated early enough, participants were aware that children could become toothless or get sick. When asked what to do if a young child's tooth hurts or is knocked out, responses included: take them to the emergency room if it happens at night, place something cold on the area, and place the tooth in a glass of milk and take it to the emergency room.

“*Cavities can form most likely during the night, especially if child sleeps with a bottle. The germs in mouth at night can form cavities.”*

“*A cavity in baby teeth can influence infection in the gums, and this can affect the adult teeth that come out.”*

“*Cavities can lead to infections in the heart and other places in the body.”*

“*Every 6 months we should take our children to the dentist. They will check with radiographs to start treating cavities early. It is important to not miss these appointments because if we miss them because we get lazy, we can be harming our child.”*

### Drinking Water/Fluoridation Knowledge

Most participants reported drinking filtered or bottled water. Some were concerned that bottled water was not good because it did not have fluoride while others were concerned about drinking water that had too much fluoride. Most did not know whether their drinking water had fluoride.

“*Don't know if our water has fluoride, and I do not know where to check.”*

“*I heard that fluoride is not good for children when they are young.”*

### Barriers to Dental Care

Most participants wanted dentists to provide them with more information about what was going to happen to their child during the dental visit. They also wanted the dentist to speak with the child to explain what would be happening to them during the visit. A few participants thought receptionists at dental clinics should be nicer to patients and more knowledgeable about insurance coverage.

“*I did not like that the dentist showed up covered in a gown with big glasses because my child got so scared. I had prepared her beforehand asking her to be ready to open her mouth big for the dentist, but when the dentist entered the room fully gowned with mask, it wasted all the preparation I did with my child. I was very frustrated about that.”*

“*I did not like that the dentists did not let me in the room when they were treating her. I did not like that they did not explain my options for treatment. I would rather that my child be put to sleep for the treatment, and even though they put her to sleep, they didn't tell me they were going to do that.”*

“*Some clinics have more experience with younger children. Some dental office will try to over diagnose or treat you. When I took my child to the dentist that didn't have a lot of experience with children, they said she needed nerve treatment and a crown. I asked why because she was only 3 years old, they said that we should go see a specialist. When we went for a second opinion with the pediatric dentist they said that all the patient needed was a small filling.”*

“*When I went to the dentists, the clinic receptionist was very mean to me. They referred me to another clinic and the number they gave me was not even to a dental clinic.”*

Focus group participants had several unanswered questions about oral health. Most questions pertained to the following topics: (1) how to properly brush and floss children's teeth and when to start using floss; (2) whether or not children should use mouthwash; (3) when should children start using toothpaste with fluoride; (4) whether teeth of children who suck their thumb can move forwards/backwards; and (5) when should a child stop breastfeeding. Results from the focus group were used to revise the COHW training curriculum.

### Phase II: COHW Training

All 13 COHWs were Latina and mostly bilingual English-Spanish. Over half (54%) were between 30 and 39 years of age (mean age was 36 years), 61% had a high school education or greater, and all had kids between the ages of 0–5 years. Most were married (69%) and homemakers (62%; see Table 2).

**Table 2:** Demographic characteristics of the 13 COHWs.

**Demographics**	***N* (%)**
**Gender**	
Female	13 (100%)
**Age**	
18–29	3 (23%)
30–39	7 (54%)
40–49	3 (23%)
**Race**	
White	13 (100%)
Ethnicity	
Latina/Hispanic	13 (100%)
**Education**	
Less than high school	5 (39%)
High school/GED	2 (15%)
Some college/college degree	5 (39%)
Post graduate/professional degree	1 (7%)
**Employment**	
Homemaker	8 (61%)
Full time worker	1 (8%)
Part time worker	4 (31%)
**Marital status**	
Married/partner	9 (69%)
Single/separated	4 (31%)
**Previous oral health worker training**	
No	12 (92%)
Yes	1 (8%)

There were significant increases in 2 of the 14 knowledge questions from pre- to post-test among COHWs in the training. The number of COHWs who knew a pregnant woman with morning sickness could protect her teeth by rinsing her mouth with water or a mixture of water and baking soda immediately after vomiting increased from *N* = 2 at pre-test to *N* = 8 at post-test (*p* = 0.05). Additionally, the number of COHWs who identified the correct age (7–9 years) at which children could generally brush their teeth well by themselves increased from *N* = 3 at pre-test to *N* = 12 at post-test (*P* = 0.05). As a composite score for all 14 knowledge questions combined for all 13 COHWs responses, the mean number of correct responses increased from 7.46 (SD 2.11) at pre-test to 10.67 (SD = 1.89) at post-test (*P* < 0.001). There were no significant increases from pre-test to post-test on the belief questions, although two showed a positive trend. The number of COHWs who believed tap water with fluoride prevents dental cavities increased from *N* = 7 at pre-test to *N* = 13 at post-test. Additionally, the number of COHWs who believed a parent's dental health affects their child's dental health increased from *N* = 7 at pre-test to *N* = 13 at post-test. Please refer to Supplementary Datasheet 1 (survey instrument) to view all knowledge and belief questions and their respective response categories.

Regarding the oral health practice questions, all 13 COHWs stated that their children had a dental home and 11 out of 13 (85%) stated the reason for their children's last visit to the dentist was routine care (dental check-up). Additionally, the COHWs reported that the mean age they took their children to the dentist for the first time was at 13 months of age (the general recommendation is to take your child to the dentist by the time the child is 12 months of age or earlier). Eight of the 13 (62%) COHWs reported they have a dental home for themselves. The COHWs who did not have a dental home for themselves stated they did not qualify for public dental insurance (*N* = 4) and one stated she had no money to pay for dental care.

### Phase III: COHW-Led Oral Health Promotion Workshops

The majority of caregivers who attended the workshops were female Latinas (88%). Just over half (51%) were between the ages of 30–39 years (mean age 36 years). Most were married (63%) and over half (56%) reported being homemakers. Approximately one-third (31%) reported a high school education or greater. The mean number of children reported by caregivers was 2.6 with a mean age of 8 years (see Table 3).

**Table 3:** Demographic characteristics of the oral health workshop attendees (*N* = 157)^*^.

**Demographics**	***N* (%)**
**Gender**	
Female	130 (88%)
Male	18 (12%)
**Age**	
18–29	26 (18%)
30–39	80 (55%)
40–49	30 (21%)
50+	9 (6%)
**Race**	
White	99 (88%)
Black/African-American	2 (2%)
Asian/Pacific islander	4 (3%)
Multi-racial	8 (7%)
**Ethnicity**	
Latino/Hispanic	150 (100%)
**Education**	
Less than high school	73 (52%)
High school/GED	35 (25%)
Some college/college degree	28 (20%)
Post graduate/professional degree	4 (3%)
**Employment**	
Homemaker	88 (58%)
Full time worker	26 (17%)
Part time worker	33 (22%)
Other	4 (3%)
**Marital status**	
Married/partner	98 (64%)
Single/separated	37 (24%)
Other	19 (12%)
**Previous oral health worker training**	
No	116 (87%)
Yes	18 (13%)

* *Frequencies do not add up N = 157 as some attendees did not answer all questions*.

Table 4 presents the pre-test and post-test comparisons for changes in five of the eight knowledge questions and all three belief questions that were statistically significant. As a composite score for all eight knowledge questions combined for all 157 caregiver responses, the mean number of correct responses increased from 2.11 (SD = 1.41) at pre-test to 3.43 (SD = 1.74) at post-test (*P* < 0.001). To see the complete list of the knowledge and belief questions with their respective response categories, please refer to the questionnaire in Supplementary Datasheet 2, 3.

**Table 4:** Changes in knowledge and beliefs from pre-test to post-tests among caregivers who attended the workshops (*N* = 157).

**Questions (with correct response category)**	**Answered correctly**		***P*-value**
	**Pre-test** ***N*** **(%)**	**Post-test** ***N*** **(%)**	
**KNOWLEDGE**			
At what age in years can children generally brush their teeth well by themselves? (7–9 years)	38 (24%)	69 (44%)	<0.05
At what age do you start using toothpaste with fluoride for your child? (6 months and/or when the first tooth comes in)	64 (41%)	121 (77%)	<0.05
Tooth decay can be prevented with (fluoride, brushing, flossing)	53 (34%)	77 (49%)	<0.05
A child's first dental visit should be (after the first baby tooth erupts or by their first birthday)	122 (78%)	143 (91%)	<0.05
When a pregnant woman has morning sickness (vomiting), what can she do to protect her teeth? (rinse mouth with water or a mixture of water and baking soda)	39 (25%)	115 (73%)	<0.05
**BELIEFS**			
Tap water is dangerous (strongly disagree/disagree)	102 (65%)	135 (86%)	<0.05
Tap water with fluoride prevents dental cavities (strongly agree/agree)	78 (50%)	143 (91%)	<0.05
A parent's dental hygiene affects their child's dental health (strongly agree/agree)	105 (67%)	143 (91%)	<0.05

When caregivers were asked, “*How often does your child eat sugary snacks like fruit snack gummies, chocolate, crackers, cookies, etc*.?,” 58% of respondents said their child eats them once a week or less than once a week. When asked, “*How many times per day does your child drink soda, fruit juice, fruit drinks, or sports drinks* ***not****with a meal?,”* 27% of the caregivers responded that their child **never** drinks soda, fruit juice, fruit drinks, or sports drinks **not** with a meal. Over three quarters of caregivers (78.4%) reported the reason for their child's last visit to the dentist was a routine check-up and only 5% reported the reason for their child's last visit to the dentist was pain. Over three quarters of caregivers (78%) said they have a dental home for themselves and 65% of them indicated it has been between 6 months and 2 years since their last dental visit. The reasons most frequently stated for not having a dental home included no dental insurance coverage (58%) and it is too expensive (21%). Still, 13% of the caregivers reported it had been 2–5 years since their own last dental visit and 2% stated they have never seen a dentist.

## Discussion

Oral health education interventions framed in a culturally appropriate and sensitive manner have a much higher likelihood of modifying participants' oral health risk behaviors than those developed generically and translated for other target populations (9, 27, 40, 41). Our study demonstrated that the use of COHWs to deliver oral health workshops to members of their community resulted in positive changes in oral health-related knowledge and beliefs following a 1 h workshop. Working with native-speakers from the community drew on the value of the community and created a comfortable and safe environment for participants to learn about oral health care.

In order to best tailor and refine the oral health curriculum content and activities for our COHW and community participants, qualitative information was gathered from a focus group which included participants with the same demographics as the target population. While the focus group participants appeared to have a fair amount of oral health knowledge, including being aware of key health promoting oral health concepts, they still had several unanswered questions about oral health care practices such as flossing, use of mouthwash, and at what age to start using toothpaste with fluoride for children. Focus group participants also reported several barriers to dental care, including lack of communication between dentist/dental office staff and the patient regarding dental procedures at the time of visit, and over diagnosing and aggressive dental treatment for young children. Dental providers who treat children should be better trained to work with children and their families. Communication strategies used by dental providers must be culturally and linguistically appropriate, and increasing the standards for dental training and practice with regards to treating young children is crucial.

The 13 COHW participating in the training showed significant increases from pre-test to post-test on the knowledge questions regarding the age when children can brush their teeth well alone and what a pregnant women with morning sickness can do to protect her teeth. Although only a few items showed statistical significance after the four training sessions, it should be noted that these 13 COHWs had a high oral health IQ before the training started. This is likely attributed to their participation in the EHS program where parents are provided with oral health information and almost half (46%) of the 13 COHWs reported a college education or greater. Thus, the lack of more statistically significant findings might be due to a ceiling effect where the COHWs had already high scores on questions at pre-test so there was little room to increase scores on the post-test.

Among the caregivers who participated in the 1 h COHW-led oral health workshops, there was a significant increase from pre- to posttest in knowledge and beliefs regarding oral health care. Significant increases in knowledge were obtained regarding when a child can brush their teeth well alone, the age when fluoridated toothpaste can be used, ways tooth decay can be prevented, when a child's first dental visit should be, and what a pregnant woman with morning sickness can do to protect her teeth. Significant positive improvements were found regarding caregiver's beliefs toward agreeing that fluoridated water can help prevent cavities, disagreeing that tap water is dangerous, and agreeing that a parent's dental health affects their children's dental health. Findings from our study are in agreement with previous findings from the Contra Caries Oral Health Education Program which found their program was effective at improving low-income Spanish-speaking parents' oral hygiene knowledge and self-reported behaviors for their young children (12).

## Limitations

This study had limitations. First and foremost, this study was not a cause and effect study. All responses to the questionnaires were self-reported and therefore subject to social desirability. This study was based on a convenience sample, thus the results may not be generalizable. Three of the COHWs training sessions were conducted by a pediatric dental resident and two nurse practitioner students. We did not calibrate the trainers, so we were not able to account for inter-trainer reliability. While the 13 COHWs who conducted the workshops with caregivers were instructed to present the same educational material, it is possible not all COHWs were 100% adherent to the program protocols since process evaluation and program monitoring activities were not incorporated into the project, thus potentially impacting the fidelity of the program. Administering the post-test immediately following the 1 h workshop limits our ability to assess the reliability of the study findings and effectiveness of the program. Additionally, the short time frame between pre-test and post-test did not allow us to assess behavior change. While increasing knowledge and changing beliefs regarding oral health care is an important step in changing behavior, it does not always translate into behavior change (42). Future studies with a longer follow up time-period (6 months−1 year) are needed to more reliably examine retention of knowledge and changes in beliefs and behaviors. Although this study did not have a control group, within-person comparisons for the pre- and post-test statistical analysis helped minimize the risk of confounding from individual characteristics and threats to validity.

## Conclusion

Few culturally and linguistically appropriate oral health promotion programs have been developed for low-income Spanish-speaking caregivers of young children (12, 37, 43–45). Our study showed that a COHW-led oral health promotion workshop resulted in significant improvements in caregivers oral health-related knowledge and beliefs. Future work using COHWs might entail implementing a formalized system of incorporating COHWs into the dental health care system. The COHWs could be responsible for connecting families with the right type of care. They could also provide referrals to patients in person and offer to assist them in setting up appointments to help ensure children actually see a provider and obtain needed follow up care, as opposed to just receiving a piece of paper with a referral written on it. Navigating the health care system is not as straightforward as many assume. Thus, viewing COHWs as an important link between the community and utilization of oral health care services, especially for high-risk and vulnerable populations, should be a priority.

Investing in COHWs to provide oral health promotion in the future will require long-term studies to validate best practice approaches that promote oral health within the context of the overall social determinants of health (access to a dental home, transportation, insurance coverage, access to healthy food options, etc.). A nationwide, streamlined, consistent training curriculum such as the American Dental Association's Community Dental Health Coordinator Curriculum (with core competencies that are evidence-based and tested) (46) as well as scope of practice and oversight will be needed. Paying COHWs through state or Los Angeles County oral health funds, Dental Transformation Initiative (DTI) pilots, and health insurance model payments should also be strongly considered.

It is crucial to design long-term *clinical* studies to examine the oral health status of the children of trained COHWs and the workshop attendees to determine if short-term improvements actually translate into positive *clinical* health outcomes in children. Previous studies have shown that caregivers have a fair amount of knowledge regarding oral health care, yet this knowledge is not necessarily translated into improvements in oral health care practices (12, 42). There continues to be a disconnect between oral health care knowledge and actual oral health care practices and clinical outcomes (similar to other chronic diseases), especially among minority and underserved populations. In light of the current high prevalence of ECC in Latino children, future studies are needed to further examine the relationship between the high prevalence of ECC among young children in underserved populations and oral health care knowledge and practices. In summary, our study results support the utilization of COHWs for ECC prevention as a viable option that must be expanded.

## Data Availability

The raw data supporting the conclusions of this manuscript will be made available by the authors, without undue reservation, to any qualified researcher.

## Ethics Statement

The study was reviewed and approved by the UCLA Office of the Human Research Protection Program for Institutional Review Board (IRB) (IRB Approval # 18-00014).

## Author Contributions

FR-G was the principal investigator and senior advisor of the study. JV was the co-principal investigator and contributed to the study design and study implementation. HA contributed to the study design, study implementation, data evaluation, and manuscript preparation. IV contributed to the study design and manuscript preparation. VK contributed to the study design and was the site cohort director. JK contributed to the manuscript preparation.

### Conflict of Interest Statement

The authors declare that the research was conducted in the absence of any commercial or financial relationships that could be construed as a potential conflict of interest.

## Supplementary Material

Supplementary Datasheet 1 Pre- and post-test for the COHWs (34 items, available in English and Spanish).

Supplementary Datasheet 2, 3 Appendix B Pre and posttest for the workshop attendees (pre:16 items and posttest: 11 items, available in English and Spanish).
