# Supplementary material for: Developing an Effective Community Oral Health Workers—“Promotoras” Model for Early Head Start
Source: Front Public Health. 2019 Jul 3;7:175. doi: 10.3389/fpubh.2019.00175 (PMC6621922; doi:10.3389/fpubh.2019.00175)
Supplement: Supplementary Datasheet 1 — Pre- and post-test for the COHWs (34 items, available in English and Spanish). [file Data_Sheet_1.PDF]

Community Oral Health Workers Project Pre/Post-test

Directions: Your answers are very important to us. Please read each question carefully and answer to the best of your ability with complete honesty. All your responses will be kept completely confidential. The following questions are about dental health. In some questions, you will be asked about what you do to take care of your child's teeth at home. For these questions, please keep in mind your youngest child.

What is the date of birth of your ***youngest child***? (Please write out):

(MM/DD/YYYY) ____ / ____ /____

Knowledge

1. Poor oral health has been linked to (circle all that apply):
   A) Diabetes
   B) Allergies
   C) Preterm babies (babies born too early)
   D) Low birth weight
   E) I don't know

2. The most common chronic childhood disease is:
   A) Asthma
   B) Hay fever/allergies
   C) Dental Cavities
   D) Heart Disease

3. What leads to dental cavities?
   A) Age
   B) Acid from bacteria
   C) Tap water
   D) Caffeine

4. At what ***age (in years)*** can children generally brush their teeth ***well*** all by themselves? ____ (fill in the blank).

5. Which liquids are okay to put in your child's bottle ***when they go to bed***?
   A) Milk
   B) Water
   C) Soda
   D) Sports drink (Gatorade, Powerade)
   E) Diluted or 100% juice

6. Caregivers can transfer bacteria/germs that cause dental caries (cavities) by (circle all that apply):
   A) Kissing their child's head
   B) Sharing eating utensils (forks, spoons, or cups)
   C) Dropping the pacifier on the floor
   D) Washing the bottles or sippy cups with the family dishes
   E) Kissing on the mouth

7. At what age do you start using toothpaste with fluoride for your child?
   A) 6 months and/or when the first tooth comes in
   B) 18 months
   C) 3 years
   E) 6 years or older

8. Dental Plaque is:
   A) A mineral that is harmless
   B) A vitamin important for healthy teeth
   C) Deposits on the teeth
   D) I don't know

9. Tooth decay can be prevented with (circle all that apply):
   A) Fluoride
   B) Brushing
   C) Vitamin C
   D) Snacking multiple times a day
   E) Flossing

10. When is it okay to give my child sweet beverages?
    A) All day or whenever they want it
    B) In between meals
    C) With meals only
    D) During naptime/bedtime

11. A child's first dental visit should be:
    A) After the first baby tooth erupts or by their first birthday
    B) When child is three years or older
    C) Only if the child has dental/mouth pain
    D) When they get their first adult tooth

12. At what age should a parent ***begin*** switching their child from a baby bottle to a cup?
    A) After 6 months of age
    B) At 12 months of age
    C) At 2 years of age
    D) No set time

13. When a pregnant woman has morning sickness (throws up), what can she do to protect her teeth right away? (circle all that apply)
    A) Brush teeth immediately
    B) Rinse mouth with plain water
    C) Rinse mouth with mouthwash
    D) Rinse mouth with a mixture of baking soda and water
    E) I don't know

14. It is safe for a pregnant woman to visit a dentist for dental treatment during pregnancy.
    A) True
    B) False
    C) I don't know

Attitude

For questions 15-24, how much do you agree or disagree with the following statements**:**

****No correct answers, preferred attitudes towards statements highlighted****

15. Poor oral health of children can lead to poor performance in school. Do you... **(circle one)**

    Strongly Agree   Agree   Disagree   Strongly Disagree   Don't know

16. A parent's dental health ***does*** affect their child's dental health. Do you… **(circle one)**

    Strongly Agree   Agree   Disagree   Strongly Disagree   Don't know

17. If my baby's pacifier falls on the ground, it is ok to clean it by putting it in my mouth before giving it back to my baby. Do you... **(circle one)**

    Strongly Agree   Agree   Disagree   Strongly Disagree   Don't know

18. Tap water is dangerous. Do you… **(circle one)**

Strongly Agree   Agree   Disagree   Strongly Disagree   Don't know

19. It is ok for me to bite off a piece of banana and then give it to my baby to eat. Do you… **(circle one)**

Strongly Agree   Agree   Disagree   Strongly Disagree   Don't know

20. Tap water with fluoride prevents dental cavities. Do you… **(circle one)**

Strongly Agree   Agree   Disagree   Strongly Disagree   Don't know

21. If my child keeps getting cavities, it's ok because they are baby teeth and will fall out anyway. Do you… **(circle one)**

Strongly Agree   Agree   Disagree   Strongly Disagree   Don't know

22. If my child keeps getting cavities, it doesn't matter because cavities can be treated with fillings and silver caps. Do you… **(circle one)**

Strongly Agree   Agree   Disagree   Strongly Disagree   Don't know

23. If my child keeps getting cavities, it's okay, all kids get cavities. Do you… **(circle one)**

Strongly Agree   Agree   Disagree   Strongly Disagree   Don't know

24. My child's diet is important for their dental health. Do you… **(circle one)**

Strongly Agree   Agree   Disagree   Strongly Disagree   Don't know

Practice

25. How often does your child eat ***sugary snacks*** like fruit snack gummies, chocolate, crackers, cookies, etc.?
A) Less than once a week
B) Once a week

C) Once a day
D) Twice a day
E) More than twice a day

26. How often do you take your child to the dentist?
    A) every few years
    B) every year
    C) every 6 months or more often
    D) only if my child has dental pain or problems

27. How many times a day do ***you*** brush your child's teeth?
    A) Not every day
    B) Once a day
    C) At least twice a day
    D) Never because my child brushes his/her own teeth

28. Do you use toothpaste ***with fluoride*** to brush your child's teeth?
    A) Yes

    B) I don't know

    C) No. If no, why not? (Please fill in below)

    ____
    ____

29. How many times per day does your child drink soda, fruit juice, fruit drinks, or sports drinks ***not with*** a meal?
    A) None
    B) 1-2 times a day
    C) 3-4 times a day
    D) 5 or more times a day
    E) Whenever they want

30. Do you have a dental office that is the usual source of dental care for ***your child***?
    A) Yes
    B) No. If no, why not? (Please fill in below)

    ____
    ____

31. What was the reason for ***your child's last*** visit to the dentist?
    A) Routine dental check-up
    B) Pain
    C) Trouble with my child's teeth
    D) Fillings, Caps, and/or removal of my child's tooth/teeth
    E) Has not seen a dentist
    F) Other (Fill in the below)
    ____
    ____

32. What was the youngest age you took your child/children to their ***first*** dental visit?
    (Please fill in below)
    ____years ____months

33. Do you have a dental office that is ***your*** regular source of dental care?
    A) Yes
    B) No. If no, why not? (Please fill in below)
    ____
    ____

34. How long has it been since ***you*** last saw a dentist?
    A) 6 months or less than 6 months
    B) 6-12 months
    C) More than 1 year ago but less than 2 years
    D) 2 years or more, but less than 5 years
    E) 5 years or more
    F) Never received dental care
