# Supplementary material for: Developing an Effective Community Oral Health Workers—“Promotoras” Model for Early Head Start
Source: Front Public Health. 2019 Jul 3;7:175. doi: 10.3389/fpubh.2019.00175 (PMC6621922; doi:10.3389/fpubh.2019.00175)
Supplement: Supplementary file 2 [file Data_Sheet_2.PDF]

Participant # ___________

Community Oral Health Workers Project Pretest

Directions: Your answers are very important to us. Please read each question carefully and answer to the best of your ability with complete honesty. All your responses will be kept completely confidential. The following questions are about dental health. In some questions, you will be asked about what you do to take care of your child's teeth at home. For these questions, please keep in mind your youngest child.

What is the date of birth of your *youngest child*? (Please write out):

(MM/DD/YYYY) ____ / ____ /____

Knowledge

1. Poor oral health has been linked to (circle all that apply):
   A) Diabetes
   B) Allergies
   C) Preterm babies (babies born too early)
   D) Low birth weight
   E) I don't know

2. At what *age (in years)* can children generally brush their teeth *well* all by themselves?
   A) 1-3 years of age
   B) 4-6 years of age
   C) 7-9 years of age
   D) 10-12 years of age

3. Caregivers can transfer bacteria/germs that cause dental caries (cavities) by (circle all that apply):
   A) Kissing their child's head
   B) Sharing eating utensils (forks, spoons, or cups)
   C) Dropping the pacifier on the floor
   D) Washing the bottles or sippy cups with the family dishes
   E) Kissing on the mouth

4. At what age do you start using toothpaste with fluoride for your child?
   A) 6 months and/or when the first tooth comes in
   B) 18 months
   C) 3 years
   E) 6 years or older

5. Tooth decay can be prevented with (circle all that apply):
   A) Fluoride
   B) Brushing
   C) Vitamin C

D) Snacking multiple times a day
E) Flossing

6. A child's first dental visit should be:
A) After the first baby tooth erupts or by their first birthday
B) When child is three years or older
C) Only if the child has dental/mouth pain
D) When they get their first adult tooth

7. At what age should a parent *begin* switching their child from a baby bottle to a cup?
A) After 6 months of age
B) At 12 months of age
C) At 2 years of age
D) No set time

8. When a pregnant woman has morning sickness (throws up), what can she do to protect her teeth right away? (circle all that apply)
A) Brush teeth immediately
B) Rinse mouth with plain water B or D, B and D
C) Rinse mouth with mouthwash
D) Rinse mouth with a mixture of baking soda and water
E) I don't know

Attitude:

For questions 9-11, how much do you agree or disagree with the following statements:

9. A parent's dental health *does* affect their child's dental health. Do you… (circle one)

Strongly Agree | Agree | Disagree | Strongly Disagree | Don't know

10. Tap water is dangerous. Do you… (circle one)

Strongly Agree | Agree | Disagree | Strongly Disagree | Don't know

11. Tap water with fluoride prevents dental cavities. Do you… (circle one)

Strongly Agree | Agree | Disagree | Strongly Disagree | Don't know

Practice

12. How often does your child eat *sugary snacks* like fruit snack gummies, chocolate, crackers, cookies, etc.?
A) Less than once a week
B) Once a week
C) Once a day
D) Twice a day
E) More than twice a day

13. How many times per day does your child drink soda, fruit juice, fruit drinks, or sports drinks *not with* a meal?
    A) None
    B) 1-2 times a day
    C) 3-4 times a day
    D) 5 or more times a day
    E) Whenever they want

14. What was the reason for *your child's last* visit to the dentist?
    A) Routine dental check-up
    B) Pain
    C) Trouble with my child's teeth
    D) Fillings, Caps, and/or removal of my child’s tooth/teeth
    E) Has not seen a dentist
    F) Other (Fill in the below)
    ____
    ____

15. Do you have a dental office that is *your* regular source of dental care?
    A) Yes
    B) No. If no, why not? (Please fill in below)
    ____
    ____

16. How long has it been since *you* last saw a dentist?
    A) 6 months or less than 6 months
    B) 6-12 months
    C) More than 1 year ago but less than 2 years
    D) 2 years or more, but less than 5 years
    E) 5 years or more
    F) Never received dental care
